# Repeat Endoscopic Ultrasound-Guided Fine-Needle Aspiration in Patients with Suspected Pancreatic Cancer: Diagnostic Yield and Associated Change in Access to Appropriate Care

**DOI:** 10.1155/2016/7678403

**Published:** 2016-08-25

**Authors:** Robert A. Mitchell, Dylan Stanger, Constantin Shuster, Jennifer Telford, Eric Lam, Robert Enns

**Affiliations:** Department of Medicine, Division of Gastroenterology, University of British Columbia, Vancouver, BC, Canada

## Abstract

*Background.* There is a high incidence of inconclusive cytopathology at initial EUS-FNA (endoscopic ultrasound-guided fine-needle aspiration) for suspected malignant pancreatic lesions. To obtain appropriate preoperative or palliative chemotherapy for pancreatic cancer, definitive cytopathology is often required. The utility of repeat EUS-FNA is not well established.* Methods*. A retrospective cohort study was conducted evaluating the yield of repeat EUS-FNA in determining a cytological diagnosis in patients who had undergone a prior EUS-FNA for diagnosis of suspected malignant pancreatic lesions with inconclusive cytopathology. The wait times to the second procedure and to decisions regarding therapy were calculated.* Results.* Overall, 45 repeat EUS-FNA procedures were performed over seven years for suspected malignant pancreatic lesions. Cytopathological class (I to IV) changed between first and second EUS-FNA in 32 patients (71%). Of 34 patients with an initially nonconclusive diagnosis, 20 had a conclusive diagnosis (59%) on repeat EUS-FNA. The cumulative yield after repeat EUS-FNA for definite pancreatic adenocarcinoma was 7 (16%). The median time interval between first and second EUS-FNA was 31 (7–175) days.* Conclusions*. A substantial number of patients had a definitive diagnosis of adenocarcinoma on repeat FNA and were, therefore, subsequently able to access appropriate care.

## 1. Introduction

Pancreatic adenocarcinoma carries a poor prognosis, with a 5-year survival rate of less than 10% [1]. The lethality of pancreatic cancer is largely due to the inherent difficulty of early detection, with only 10–20% of patients being diagnosed at a stage of resectable disease [1, 2]. To guide management decisions, EUS guided FNA (EUS-FNA) to obtain a tissue diagnosis is the standard of care for potential pancreatic malignancies [3].

EUS has become vital in the diagnosis of pancreatic cancer. Studies have demonstrated that EUS is more sensitive than both transabdominal US and CT scan in detecting solid pancreatic masses, and EUS-FNA has emerged as an essential diagnostic tool for pancreatic cancer [4, 5]. A recent meta-analysis from pooled studies demonstrated that the sensitivity of EUS-FNA in the correct diagnosis of a solid pancreatic mass is 86.8% [6]. It has been reported to range between 62% and 96% in individual studies [7–9]. EUS-FNA is therefore the test of choice for histology in potential pancreatic cancer.

Despite the demonstrated diagnostic sensitivity of EUS-FNA, there is a relatively high rate of inconclusive diagnoses resulting from the initial EUS-FNA, at 6%–11% [10, 11]. An inconclusive result on initial FNA can potentially delay diagnosis and access to appropriate care. In an effort to improve diagnostic yield, many patients undergo a repeat EUS-FNA, and recent studies have demonstrated that a repeat EUS-FNA can increase diagnostic accuracy and potentially alter the clinical diagnosis in patients with pancreatic lesions [12–18].

In many Canadian jurisdictions, access to appropriate therapy for initial management of pancreatic cancer requires a cytopathological diagnosis of pancreatic adenocarcinoma (class IV or V cytology on FNA as per Papanicolaou class [19]). It is, therefore, necessary to clarify how an appropriate diagnosis can be reached in patients with a high clinical probability of pancreatic cancer where a diagnosis remains unclear after an initial EUS-FNA. It is not known to what extent a second procedure may delay eventual therapy in patients with a pancreatic lesion suspicious for adenocarcinoma.

Although there are some studies evaluating a second biopsy in the setting of pancreatic lesions, the yield of repeat EUS-FNA and its effect in terms of delay to treatment or treatments offered has not been studied. This study aims to report the outcomes of repeat EUS-FNA for Canadian patients with suspected pancreatic cancer and to evaluate how access to therapies for pancreatic cancer is influenced by a repeat procedure.

## 2. Methods

### 2.1. Patient Selection

This retrospective cohort study was conducted at St. Paul's Hospital in Vancouver, British Columbia. St. Paul's Hospital is a tertiary referral center in downtown Vancouver. Two trained and experienced therapeutic endoscopists in the gastroenterology department perform EUS-FNA. All patients in this study were selected from a database of patients who had undergone repeat EUS-FNA for investigation of a pancreatic mass between 2007 and 2014. The total number of individual patients undergoing EUS for all indications (not exclusively for investigations of pancreatic masses) at St. Paul's Hospital over the study period was 3035. These patients were identified from billing codes. The total number of individual patients undergoing a second EUS for any indication was 182 over the study period. The total number of patients undergoing a second EUS-FNA for investigation of a pancreatic mass over the study period was 45. The indication for EUS-FNA was determined from clinic notes. Clinical presentation and location of lesions leading to the first EUS-FNA are described further in* Results*. All patients included in the study underwent repeat EUS-FNA for investigation of a pancreatic mass suspicious for malignancy, after initial EUS-FNA results demonstrated inconclusive pathology (indeterminate: class I, negative: class II, and atypical cells: class III). Four patients with likely pancreatic adenocarcinoma (class IV pathology) on first EUS-FNA underwent repeat EUS-FNA, the reasons for which are described further in* Results*. Sequential patients who had undergone a repeat procedure for this indication during the study period were included in the study. All patients provided written informed consent to undergo both the initial and the repeat procedures. This study was approved by an Institutional Review Board at the University of British Columbia.

### 2.2. Data Collection

Clinical data was obtained from patients' operative notes, clinical notes, and referral letters. Pathological data was obtained from pathology reports for each patient—cytology was obtained from final pathological reports reported by attending pathologists at St. Paul's Hospital. Cytology was coded according to pathological class as follows: indeterminate (class I), negative for malignancy (class II), atypical cells (class III), likely pancreatic adenocarcinoma (class IV), diagnostic for pancreatic adenocarcinoma (class V), neuroendocrine tumor (NET), or lymphoma [19]. Data regarding operative techniques for initial and repeat procedure were obtained from operative reports. Follow-up data was completed through May 2015 ensuring at least one-year clinical follow-up for all patients. Mortality was determined from clinical records and hospital database. Complications were defined as negative outcomes reported by the patient, or treating physician which were felt to be related to the procedure, and not part of the usual clinical course of postprocedure recovery. Data regarding complications were obtained from clinic notes and discharge summaries.

### 2.3. EUS-FNA Procedure

All procedures were performed by two trained and experienced therapeutic gastroenterologists. EUS-FNA was performed using a linear echoendoscope, with various needles and calibers (reported in* Results*). Doppler sonography was used to identify vascular structures. The number of passes was not specifically recorded in this study and varied among patients. In general, multiple passes were made (typically 3-4 per mass). FNA technique varied between gastroenterologists. Generally, the needle was inserted into the target lesion with the stylet inserted. The stylet was then removed and to-and-fro motions were made with or without suction, or the stylet was extracted slowly while to-and-fro motions were made within the lesion. Over the study period, the method for cytopathological preparation changed from placement of the specimen on air-drying slides with subsequent preparation and analysis by on-site cytology technologist to placement of the specimen directly into CytoLyt® (Hologic, Bedford, MA, USA) fixative. The specimens were then transported to the hospital laboratory where they were prepared and evaluated by a pathologist.

### 2.4. Statistical Analysis

Continuous variables were reported as median (range) and discrete variables were expressed as *n* (%) unless otherwise specified. Data analysis was conducted using statistical software (SPSS Statistics® v22, IBM, Armonk, NY, USA). Fisher's exact test was used to compare categorical variables. Student's *t*-test was used to compare continuous variables. *P* values were calculated as 2-tailed and a value of ≤0.05 was interpreted as significant.

## 3. Results

### 3.1. Patient Characteristics

Forty-five individual patients underwent repeat EUS-FNA of a suspected pancreatic adenocarcinoma during the study period. The mean patient age was 67.3 years (±9.5 years), and the majority of study subjects were male (62%, *n* = 28). Symptoms reported at initial presentation included abdominal pain (56%, *n* = 25), jaundice (27%, *n* = 12), and weight loss (20%, *n* = 9). In 12 patients, pancreatic cancer was suspected on the basis of incidental imaging findings (27%). The location of the mass was the pancreatic head/uncinate in 34 patients (75%), pancreatic tail in seven (16%), and pancreatic body in four (9%). The mean lesion size in greatest axis was 2.6 cm (±1.3 cm).

### 3.2. Yield of Repeat EUS-FNA

Cytopathological class (I to IV) changed between first and second FNA in 32 patients (71%) (Table 1). Seven patients received a definitive diagnosis with the repeat EUS-FNA. Of the 34 patients with indeterminate or atypical cytology at initial exam, 7 (21%) were diagnosed with or had likely pathology for pancreatic cancer, 1 (3%) with lymphoma, 1 (3%) with NET, and 11 (32%) with benign disease, while 14 (41%) still had an indeterminate or atypical cytology on the repeat FNA. Therefore, of 34 patients with nonconclusive pathology on initial EUS-FNA, 20 patients had conclusive pathology on repeated EUS-FNA (59%). Pancreatic adenocarcinoma was definitively diagnosed in seven patients on repeat FNA (16%), all of whom had nondiagnostic pathology prior to repeat study. Four patients who had initial EUS-FNA showing likely pancreatic adenocarcinoma had a repeat FNA because of the following. (1) Disagreement occurred between the five pathologists reviewing the sample. Some favored likely malignancy, while others felt changes reflective of inflammation; therefore a repeat procedure was performed. (2) Repeat confirmatory EUS-FNA was requested from treating surgeon due to uncertainty of diagnosis on imaging, and extent of surgery was required. (3) It was likely adenocarcinoma on first biopsy, but it was unclear if NET or adenocarcinoma based on first EUS-FNA. Cancer agency requested repeat EUS-FNA prior to initiating treatment. (4) It was likely adenocarcinoma on first biopsy; however possibility of inflammatory process raised by pathologist. The patient was referred to the Cancer Agency, and additional tissue was requested before proceeding with chemotherapy. Three of these patients had diagnostic (class V) pathology on repeat EUS-FNA, while one had indeterminate pathology on repeat EUS-FNA. A diagnosis of negative pathology for malignancy was made in 16 cases on repeat FNA, with 11 of these being new classifications made on repeat FNA, all from previously indeterminate or atypical samples. The most common needle size used for repeat FNA was 22-gauge, which was used in 35 repeat procedures (78%), followed by 25-gauge used in 8 repeat procedures (18%); 19-gauge was used in 2 cases (4%).

### 3.3. Complications

There were no definite complications associated with a repeat EUS-FNA. There were two emergency room presentations in patients with a recent repeat EUS-FNA: one was abdominal pain of unknown etiology in a patient presenting the emergency department five days after repeat EUS-FNA; the second was a nonspecific and unrelated presentation to the emergency department one day after repeat EUS-FNA. Neither presentation to the emergency department resulted in a hospital admission or a discernable change in management.

### 3.4. Access to Care

Treatment was assessed following second EUS-FNA (Table 2). A total of 16 patients (36%) were offered treatment after a repeat EUS-FNA. Five patients were offered chemotherapy (11%) and 11 patients were offered surgery (24%) (Table 3). The median time from referral of the patient for assessment to first EUS-FNA was 14 days (1–200 days); median time from initial referral to second EUS was 48 days (11–212 days). The median interval delay from first to second EUS was 31 days (7–175 days). The median time from referral for FNA to treatment (chemotherapy or surgery) was 121 days (33–389 days) (Table 4). In the total group of patients followed, 33 survived to one year (73%). Two-year follow-up data was available for 42 of 45 patients. Among the 42 patients with two-year follow-up data available, 20/42 patients (48%) were alive at two years. Four of 10 patients with a likely or definitive diagnosis of adenocarcinoma on repeat procedure were offered treatment (40%), compared to 12 of 35 patients (34%) who were offered treatment with a repeat FNA that was not suggestive of pancreatic adenocarcinoma (*P* = 0.73). Nine patients without evidence of adenocarcinoma on repeat biopsy were offered surgery. This was because of a third EUS-FNA demonstrating adenocarcinoma (1), a high degree of suspicion for cancer but technically difficult EUS-FNA leading to laparotomy diagnostic for adenocarcinoma (1), ongoing suspicion for malignancy due to progression of mass on imaging with worsening symptoms (5), and the presence of a NET on second EUS-FNA (2). Chemotherapy was offered to three patients without evidence of adenocarcinoma on second EUS-FNA. This was because of enlargement and mass effect of a lymph node felt to be metastatic pancreatic cancer (1), a third EUS-FNA diagnostic of malignancy (1), and enlargement of pancreatic mass on imaging, plus ongoing symptoms in patient (1). Ten of 35 patients (29%) with indeterminate, negative, or atypical pathology underwent a third EUS-FNA at our site during the follow-up period. At one year, among patients with a likely or definitive diagnosis of pancreatic adenocarcinoma on repeat FNA (class IV or V), 6 of 10 (60%) had survived, compared with 27 of 35 (77%) patients with a repeat FNA that was not diagnostic of pancreatic adenocarcinoma (*P* = 0.42). Two-year survival data was available for 42 of 45 patients (three patients with recruitment from May 2013 to May 2014 and therefore incomplete two-year follow-up). At two years, among patients with a likely or definitive diagnosis of pancreatic adenocarcinoma on repeat FNA (class IV or V), 1 of 9 had survived (11%), compared with 19 of 33 (58%) patients with a repeat FNA that was not diagnostic of pancreatic adenocarcinoma (*P* = 0.02) (Table 5).

### 3.5. Final Diagnosis in Patients with Indeterminate, Negative, or Atypical Pathology on Repeat EUS-FNA

Additional information was gathered regarding the ultimate diagnoses for patients with indeterminate (class I), negative (class II), or atypical (class III) pathology on second EUS-FNA.

Of seven patients with a second EUS-FNA demonstrating indeterminate (class I) pathology, there was detailed follow-up information available for seven patients (100%). One patient underwent an open biopsy demonstrating diffuse large B-cell lymphoma. Two patients underwent third EUS-FNA that were again indeterminate; masses in both cases were ultimately thought to be benign and cystic. One patient developed gastric outlet obstruction as a result of the pancreatic mass and died from complications of this; a tissue diagnosis was never made. Two patients had third EUS-FNA demonstrating likely (class IV) pancreatic cancer. One patient had repeat EUS-FNA again indeterminate; however PET scan demonstrated metastatic disease and patient died before a tissue diagnosis was made.

Of 16 patients with a second EUS-FNA demonstrating negative (class II) pathology, there was detailed follow-up information available for ten patients (62%). Four patients were ultimately diagnosed with pancreatic cancer. One patient had an open biopsy and was diagnosed with metastatic renal cell carcinoma. Two patients had repeated EUS-FNA that were again negative and did not receive clear diagnoses at last follow-up. Two patients were diagnosed with pancreatitis. One patient was diagnosed with a pancreatic cyst. Of the patients with a second EUS-FNA that was negative (class II) and who subsequently died, information on cause of death was available for four patients, and in all four patients, cause of death was attributed to complications of pancreatic cancer. In one of these four patients, diagnosis of pancreatic cancer was made on repeated EUS-FNA; the remainder were diagnosed by imaging and clinical suspicion. One of these patients underwent surgical resection, which demonstrated pancreatic adenocarcinoma.

Of eight patients with a second EUS-FNA demonstrating atypical (class III) pathology, there was detailed follow-up information available for six patients (75%). One underwent Whipple resection and was diagnosed with metastatic RCC. One patient underwent repeated EUS-FNA demonstrating negative (class II) pathology and one was with likely (class IV) pathology. One patient had an open biopsy demonstrating NET. Three patients were ultimately diagnosed with pancreatic adenocarcinoma: one by repeated EUS-FNA and two based on imaging findings and clinical suspicion.

## 4. Discussion

Studies have consistently demonstrated the diagnostic utility of repeat EUS-FNA in patients where a diagnosis of pancreatic cancer is strongly suspected, but initial EUS-FNA is negative or nondiagnostic [12–18]. This report confirmed these findings in a Canadian patient population. This study demonstrated that repeat FNA yields an altered diagnosis in 71% of patients. This is similar to previous studies, where the ability of a second EUS-FNA to alter initial diagnosis has been reported to range from 63% [14] to 82% [13]. As in previous research studies on this topic, the reason for repeated EUS-FNA in our study was uniformly due to inconclusive pathology on initial EUS-FNA in patients with a high degree of clinical suspicion for pancreatic cancer (indeterminate, negative, or atypical cytopathology) [13]. Importantly, 59% of patients with initially nonconclusive pathology (indeterminate or atypical) have conclusive pathology (benign, likely adenocarcinoma, definitive adenocarcinoma, NET, and lymphoma) on repeat EUS-FNA, while 31% of patients in this study had a likely diagnosis of pancreatic adenocarcinoma or other malignancy (class IV or V pathology, NET, and lymphoma) on repeat EUS-FNA, with 24% of patients having a definite diagnosis (class V pathology, NET, and lymphoma). This is in keeping with other studies, where the ability of repeat EUS-FNA to definitively diagnose malignancy has been reported between 21% [18] and 46% [12].

The primary aim of this study was to explore how repeat FNA facilitated access to treatment for pancreatic adenocarcinoma. Repeat EUS-FNA is often used in a specific patient population where the pretest likelihood of cancer is high enough to warrant a second diagnostic procedure, but not high enough to proceed directly to surgery [20]. Patients who are not candidates for adjunctive therapy or surgery might be followed clinically and managed supportively rather than considered for a repeat biopsy. In our jurisdiction, a positive cytopathological result is required to access chemotherapy or radiation therapy and is usually requested before surgery. Additionally, as demonstrated in the present study, a repeat EUS-FNA may occasionally be requested by treating surgeons or oncologists in patients where initial biopsy demonstrates likely adenocarcinoma (class IV), but there is some degree of uncertainty in the diagnosis, or if a particularly morbid therapy is planned. In the current study, we demonstrated that patients have a median delay of 31 days from initial to repeat FNA and thus a significant delay to receiving eventual treatment. Other studies have reported a similar interval from first to second procedure of 33 days [18]. This delay may be secondary to time required to review the sample and occasionally obtain second opinions on available histology. There may also be a time delay associated with multidisciplinary discussions that are held with surgeons and oncologists after final histology from the first EUS has been obtained. Unfortunately the exact reasons for delay between first and second EUS-FNA are not known in this population and this is an interesting area of potential future research.

Treatment was offered to relatively few patients in the total patient group and was offered at similar rates between those patients that had adenocarcinoma likely or diagnosed on repeat FNA (class IV or V) and those who did not. The low observed rates of offered treatment in this population are in keeping with the general rates of treatment in newly diagnosed pancreatic cancer and likely due to the cancer stage at diagnosis or the poor functional status of these patients [21]. Staging information at the time of diagnosis and performance status was not recorded in this study. The relatively high rates of offered treatment in patients without pancreatic adenocarcinoma on repeat EUS-FNA are striking and seem to indicate an eventual willingness to offer treatment to these patients without definitive adenocarcinoma on cytopathology. The reasons for this are described in Results but are often related to progression of the mass on imaging or progression of symptoms.

If the goal of repeat EUS-FNA is to facilitate access to care, one would hope to observe more treatments offered to those with a definitive cytopathological diagnosis made on repeat FNA than to patients with negative or inconclusive results on repeat EUS-FNA. Patients in this study waited a median of 31 days for a repeat procedure with the goal of achieving definitive cytopathological diagnosis and were not offered surgery or chemotherapy at a significantly different rate than those without cytopathology suggestive of cancer. Additionally, a repeat diagnostic procedure is not without risk and can be associated with complications that in this study population were minor, but in other larger studies have been important [22]. Of note, this study only measured management related to further chemotherapy, radiation therapy, or surgery. There are other important management decisions (including referral to palliative care, support groups, and other end-of-life planning) that were not measured in this study. Additionally, with new and potentially more expensive chemotherapeutic options and different management styles emerging, the importance of definitive diagnosis may become more critical, and rapid access to obtaining this tissue becomes more critical.

Other limitations of this study include the small sample size and the relatively few number of patients in the group with a diagnosis of adenocarcinoma (class IV or V pathology). Different pathologists reviewed initial and repeat pathology, and there may be interreviewer variability in the interpretation of cytopathology. Given the small number of patients in this study, this is an important limitation of these results. Also, this data is from a single center, and it is possible that varying practices exist elsewhere with respect to offering treatment to patients with suspected pancreatic cancer in the absence of definite cytopathology. Additionally, since this was a single-center study, there were likely patients who underwent a first EUS-FNA at our center with inconclusive pathology and who went on to have a second EUS-FNA at a different center and therefore follow-up details were not included in this study. This decreases the overall number of patients in the present study and may bias the study sample to those that were well enough to travel to our site for their second EUS-FNA or be seen in follow-up. Unfortunately, few of the study patients ultimately underwent surgical resection and therefore do not have final surgical pancreatic pathology. As such, we cannot comment on the sensitivity and specificity of repeat EUS-FNA in this population. Further investigation in this study population is warranted to directly compare meaningful clinical endpoints and treatment rates between patients with an uncertain initial FNA who do and do not undergo a repeat EUS-FNA.

In conclusion, repeat EUS-FNA can alter the pathologic diagnosis and lead to a definitive diagnosis in many patients with pancreatic adenocarcinoma. In this patient population, however, this did not lead to a significant increase in the rate of treatment that was offered or administered and was associated with a substantial delay in time between first and second EUS-FNA.

## Figures and Tables

**Table 1:** Cytopathological class of first and second EUS-FNA in patients who underwent repeat EUS-FNA for diagnosis of suspected pancreatic malignancy at St. Paul's Hospital, Vancouver, British Columbia, between 2007 and 2014.

	Cytopathological class	Second EUS-FNA							Total
		Indeterminate (I)	Negative (II)	Atypical (III)	Likely (IV)	Diagnostic (V)	NET	Lymphoma	
First EUS-FNA	Indeterminate (I)	5	8	6	1	2	0	0	22
	Negative (II)	0	5	0	0	0	0	0	5
	Atypical (III)	1	3	2	2	2	1	1	12
	Likely (IV)	1	0	0	0	3	0	0	4
	Diagnostic (V)	0	0	0	0	0	0	0	0
	NET	0	0	0	0	0	1	0	1
	Lymphoma	0	0	0	0	0	1	0	1

	Total	7	16	8	3	7	3	1	45

Comparison of first and second EUS-FNA results in patients with suspected pancreatic cancer. Divided by cytopathological diagnosis (EUS-FNA: endoscopic ultrasound fine-needle aspiration; NET: neuroendocrine tumor).

**Table 2:** Treatment and wait times divided by result of second EUS-FNA in patients who underwent repeat EUS-FNA for diagnosis of suspected pancreatic malignancy at St. Paul's Hospital, Vancouver, British Columbia, between 2007 and 2014.

Cytopathology on 2nd EUS-FNA	Patients (*n* = 45)	Surgery performed	Chemotherapy started	Wait time to surgery or chemotherapy (days)	Interval between 1st and 2nd EUS-FNA (range)
Indeterminate	7 (15%)	0	0	—	28 (24–175)
Negative	16 (35%)	1	1	94, 148	39 (18–127)
Atypical	8 (18%)	1	1	36, 231	20 (9–37)
Likely	3 (7%)	2	0	33, 200	76 (16–80)
Diagnostic	7 (16%)	0	2	48, 265	19 (7–56)
NET	3 (7%)	2	0	84, 389	51 (28–56)
Lymphoma	1 (2%)	0	0	—	13

Treatment decisions and associated wait times following second EUS-FNA in patients with suspected pancreatic cancer (NET: neuroendocrine tumor; EUS-FNA: endoscopic ultrasound fine-needle aspiration).

**Table 3:** Treatment details of all patients following repeat EUS-FNA for diagnosis of suspected pancreatic malignancy at St. Paul's Hospital, Vancouver, British Columbia, between 2007 and 2014.

Treatment	Patients (*N* = 45)
	*n* (%)
*Chemotherapy*	
Yes	4 (9)
No	40 (89)
Offered & declined by patient	1 (2)
*Surgery*	
Yes	6 (13)
No	34 (76)
Offered & declined by patient	5 (11)

Details of treatment in 45 patients with suspected pancreatic cancer following a second EUS-FNA procedure (EUS-FNA: endoscopic ultrasound fine-needle aspiration).

**Table 4:** Time to first and second EUS-FNA and eventual treatment among patients with suspected pancreatic malignancy at St. Paul's Hospital, Vancouver, British Columbia, between 2007 and 2014.

	Days (range)
Time from referral to 1st EUS-FNA	14 (1–200)
Time from referral to 2nd EUS-FNA	48 (11–212)
Interval time from 1st to 2nd EUS-FNA	31 (7–175)
Time from referral to chemotherapy or surgery	121 (33–389)

Time to treatment from referral to first and second EUS-FNA or to treatment in 45 patients with suspected pancreatic cancer (EUS-FNA: endoscopic ultrasound fine-needle aspiration).

**Table 5:** Comparison of treatment and survival between patients by cytopathologic classes of repeat EUS-FNA for suspected pancreatic malignancy at St. Paul's Hospital, Vancouver, British Columbia, between 2007 and 2014.

	Repeat EUS-FNA demonstrating definite or likely adenocarcinoma (*n* = 10)	Repeat EUS-FNA not demonstrating definite or likely adenocarcinoma (*n* = 35)	*P* value
Offered treatment, *n* (%)	4/10 (40%)	12/35 (34%)	0.73
One-year survival, *n* (%)	6/10 (60%)	27/35 (77%)	0.42
Two-year survival, *n* (%)^+^	1/9 (11%)	19/33 (56%)	0.02^*∗*^

Treatment and survival and one and two years among 45 patients with suspected pancreatic cancer who underwent repeat EUS-FNA (EUS-FNA: endoscopic ultrasound fine-needle aspiration; SD: standard deviation; *∗* indicates statistical significance, *P* < 0.05; + calculated only for 42/45 patients recruited into the study prior to May 2013.).
